# Dynamic Change of Gut Microbiota During Porcine Epidemic Diarrhea Virus Infection in Suckling Piglets

**DOI:** 10.3389/fmicb.2019.00322

**Published:** 2019-02-25

**Authors:** Anni Huang, Rujian Cai, Qun Wang, Lei Shi, Chunling Li, He Yan

**Affiliations:** ^1^School of Food Science and Engineering, South China University of Technology, Guangzhou, China; ^2^Institute of Animal Health, Guangdong Academy of Agricultural Sciences, Guangzhou, China; ^3^Guangdong Provincial Key Laboratory of Livestock Disease Prevention, Guangzhou, China; ^4^Guangdong Open Laboratory of Veterinary Public Health, Guangzhou, China; ^5^Institute of Food Safety and Nutrition, Jinan University, Guangzhou, China

**Keywords:** gut microbiota, PEDV infection, suckling piglets, SCFA-producing bacteria, core microbiota

## Abstract

Porcine epidemic diarrhea (PED) is a disease that has a devastating effect on livestock. Currently, most studies are focused on comparing gut microbiota of healthy piglets and piglets with PED, resulting in gut microbial populations related to dynamic change in diarrheal piglets being poorly understood. The current study analyzed the characteristics of gut microbiota in porcine epidemic diarrhea virus (PEDV)-infected piglets during the suckling transition stage. Fresh fecal samples were collected from 1 to 3-week-old healthy piglets (*n* = 20) and PEDV infected piglets (*n* = 18) from the same swine farm. Total DNA was extracted from each sample and the V3–V4 hypervariable region of the 16S rRNA gene was amplified and sequenced using the Illumina MiSeq platform. Statistically significant differences were observed in bacterial diversity and richness between the healthy and diarrheal piglets. Principal coordinates analysis (PCoA) showed structural segregation between diseased and healthy groups, as well as among 3 different age groups. The abundance of *Escherichia-Shigella*, *Enterococcus*, *Fusobacterium*, and *Veillonella* increased due to dysbiosis induced by PEDV infection. Notably, there was a remarkable age-related increase in *Fusobacterium* and *Veillonella* in diarrheal piglets. Certain SCFA-producing bacteria, such as *Ruminococcaceae_UCG-002*, *Butyricimonas*, and *Alistipes*, were shared by all healthy piglets, but were not identified in various age groups of diarrheal piglets. In addition, significant differences were observed between clusters of orthologous groups (COG) functional categories of healthy and PEDV-infected piglets. Our findings demonstrated that PEDV infection caused severe perturbations in porcine gut microbiota. Therefore, regulating gut microbiota in an age-related manner may be a promising method for the prevention or treatment of PEDV.

## Introduction

Porcine epidemic diarrhea (PED) is a common enteric infection caused by the porcine epidemic diarrhea virus (PEDV) (Song et al., 2015). PED presents with various clinical symptoms, including acute diarrhea, vomiting, anorexia, dehydration and weight loss (Gallien et al., 2018). PEDV may infect pigs of any age, ranging from neonates to sows or boars. However, the severity of PED in pigs differs according to age (Shibata et al., 2000). In fact, mortality caused by PED is close to a 100% for newborn piglets and may be as high as 80% for suckling piglets, resulting in serious losses to the swine industry (Mole, 2013).

Gut microbiota harbors trillions of microbes providing many biological functions to the host (Bäckhed et al., 2007; Chen et al., 2017). Intestinal microbiota assists in maintaining normal functioning of the intestinal mucosal barrier and contributes to the development of the host immune system (Fernández et al., 2003; Ivanov et al., 2009; Garrett et al., 2010). Increasingly, evidence indicates that gut microbiota may play a crucial role in the regulation, elimination and potentiation of infectious diseases (McKenney and Pamer, 2015; Niederwerder, 2017). For many years, studies investigating the relationship between gut microbiota and disease have focused on pathogens (Niederwerder, 2017). However, age is also a very important factor in shaping gut microbiota (Shibata et al., 2000). In pigs, dynamic changes in the percentage of gut microbiota were observed at different growth stages and under different conditions (Park et al., 2014; Kim et al., 2015).

Previous studies have shown that PEDV may induce an imbalance in the gut microbiota of piglets and sows, leading to a reduction in commensal bacteria, and an increase in pathogenic bacteria (Kou et al., 2015; Liu S. et al., 2015; Song et al., 2017; Huang et al., 2018). However, characterization of gut microbiota in PEDV-infected piglets at different growth stages has been limited. The current study compared gut bacterial communities of healthy piglets with those affected with PED, and evaluated dynamic changes in the gut microbiota of PEDV-infected piglets.

## Materials and Methods

### Animals and Sample Collection

This trial was conducted at a commercial swine farm located in Guangdong province, Southern China. In April 2017, severe diarrhea in sucking piglets was discovered in some pens. Fecal samples were taken from 38 piglets at 1–3 weeks of age before weaning from this swine farm, including 18 piglets infected with PEDV (*n* = 6 piglets per week) and 20 healthy piglets. All samples were tested using Antigen Rapid PED, TGE and Rota Ag Test Kit (Bionote, Hwaseong, South Korea) and the diarrheal samples were found to be positive for the PEDV antigen. Positive samples were confirmed by a PEDV-transmissible gastroenteritis virus (TGEV)-porcine rotavirus triple real-time RT-PCR kit. All 38 fresh fecal samples were immediately frozen in liquid nitrogen following collection and stored at -80°C until extraction of genomic microbial DNA. This work complied with the Laboratory Animals-Guideline of Welfare and Ethics published by the General Administration of Quality Supervision, Inspection, and Quarantine of the People’s Republic of China. The animal experimental proposals were approved by the Animal Care and Use Committee of South China University of Technology and were in compliance with the Ethical Committee of South China University of Technology, Guangzhou, China.

### DNA Extraction

Total DNA was extracted from the fecal samples using QIAamp DNA Stool Mini Kit (QIAGEN, Germany) according to manufacturer’s protocol. The final DNA concentration and purity were determined using a NanoDrop 2000 UV-vis spectrophotometer (Thermo Scientific, Wilmington, MA, United States), and DNA quality was evaluated by 1% agarose gel electrophoresis.

### Polymerase Chain Reaction Amplification and Sequencing

Polymerase chain reaction (PCR) amplification was performed in triplicate for each fecal sample in total reaction volumes of 20 μL containing 4 μL of 5× FastPfu Buffer, 2 μL of 2.5 mM dNTPs, 0.8 μL of each primer (5 μM), 0.4 μL of FastPfu Polymerase, and 10 ng of template DNA. The PCR primers flanked the V3–V4 hypervariable region of the bacterial 16S rRNAs and included primes 338F (5′-ACTCCTACGGGAGGCAGCAG-3′) and 806R (5′-GGACTACHVGGGTWTCTAAT-3′). PCR thermocycling conditions were denaturation at 95°C for 3 min, followed by 27 cycles at 95°C for 30 s, annealing at 55°C for 30 s, elongation at 72°C for 45 s and a final extension at 72°C for 10 min. The resulting PCR products were confirmed by 2% agarose gel electrophoresis, purified using an AxyPrep DNA Gel Extraction Kit (Axygen Biosciences, Union City, CA, United States) and quantified using QuantiFluor^TM^-ST (Promega, Madison, WI, United States), according to the manufacturer’s protocol. Based on the standard protocols of Majorbio Bio-Pharm Technology Co., Ltd. (Shanghai, China), PCR-purified amplicons were pooled in equimolar amounts and paired-end sequenced (2 × 300 bp) on a MiSeq platform (Illumina, San Diego, CA, United States). The 16S rRNA gene sequence information contained in this paper was deposited in the GenBank Sequence Read Archive database under accession number SRP172711.

### Processing of Sequencing Data

To minimize the effects of random sequencing errors, raw fastq files were demultiplexed, quality-filtered using Trimmomatic, and merged via FLASH using the following criteria: (i) primers matched exactly, only two nucleotide mismatches were allowed, and reads containing ambiguous bases were removed; (ii) reads were truncated at any site receiving an average quality score less than 20 over a 50-bp sliding window; and (iii) sequences with an overlap greater than 10 bp were merged according to their overlap sequence.

In addition, following identification and removal of chimeric sequences using UCHIME^1^, sequences with ≥97% similarity were assigned to the same operational taxonomic units (OTUs) using UPARSE version 7.1^2^. A representative sequence for each OTU was screened for further annotation. Each 16S rRNA gene sequence was taxonomically analyzed against the Silva (SSU123) 16S rRNA database, using the RDP Classifier algorithm^3^ with a confidence threshold of 70%.

### Statistical Analysis

Alpha diversity metrics of different groups were compared using Wilcoxon signed-rank test and adjusted for false discovery rate. Statistical significance was set at *p* < 0.05. Statistical comparisons of unweighted UniFrac distances among groups were performed via analysis of similarities (ANOSIM). ANOSIM was performed using the “vegan” package of R (v3.0.3). Heatmaps were generated with the R-package gplots at the genus level. A colinear relation diagram was generated using the Circos-0.67-7 package^4^. COG (Clusters of Orthologous Groups) category assignments were performed through BLAST-based similarity searches to identify the closest matching sequence in the STRING database (Search Tool for the Retrieval of Interacting Genes^5^) (*E*-value < 10^-6^).

## Results

### Characteristics of Sequencing Data

We collected a total of 1,616,525 quality-filtered and chimera-checked sequences with an average length of 440.25 bp across all samples. The mean number of reads per sample was 42,540, ranging from 31,732 to 56,822 reads. Good’s coverage ranged from 99.86 to 99.97%, indicating that sequencing accuracy was reliable. Multiple rarefaction curves were measured using several metrics, namely Shannon, Simpson, Chao1, and Sobs, which confirmed adequate sequence coverage for all samples (Supplementary Figure S1).

**FIGURE 1:**
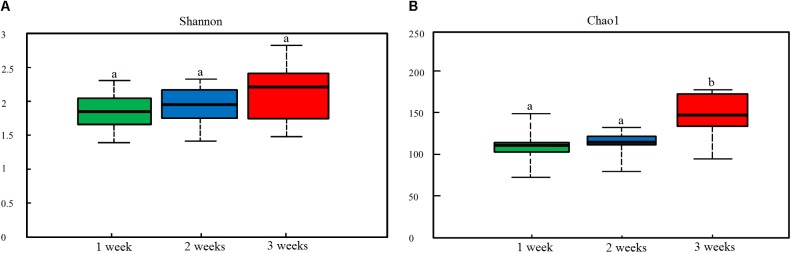
Dynamic changes in gut microbial alpha diversity of PEDV-infected piglets determined by **(A)** Shannon index and **(B)** Chao1 index. Different letters above the bars denote a significant difference in alpha diversity index among the groups tested by paired sample Wilcoxon signed-rank test and adjusted for false discovery rate (FDR, *p* < 0.05).

To characterize the levels and patterns of diversity within individuals, different measures of alpha diversity were applied. The Shannon and Chao1 indices were higher in the healthy individuals compared with those infected with PED (*p* < 0.05) (Supplementary Figure S2). Figure 1 indicated the specific bacterial indices (Shannon and Chao1) made from diarrheal piglets of different age groups. There were no statistically significant differences in bacterial diversity and richness among the 3 age groups (*p* > 0.05), except for bacterial richness measured via Chao1 2 – 3 weeks after birth (*p* < 0.05).

**FIGURE 2:**
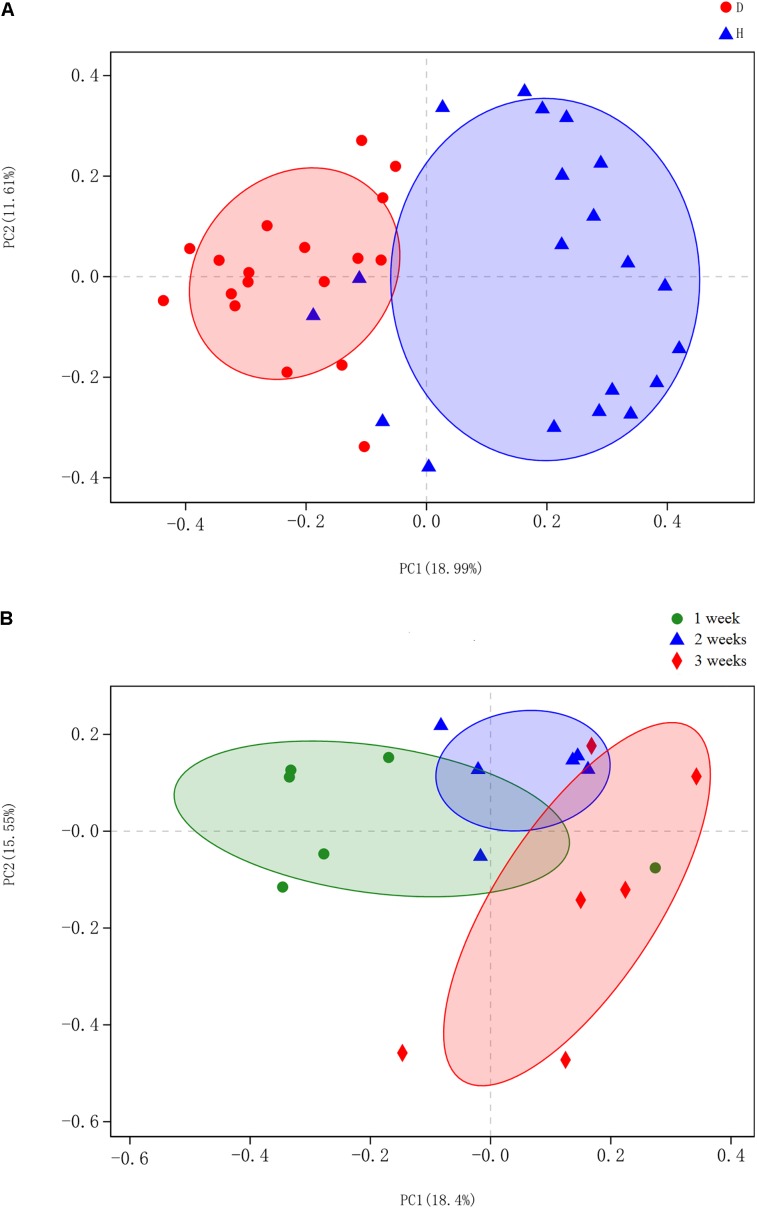
Principal coordinates analysis (PCoA) was performed at the operational taxonomic unit (OTU) level based on unweighted UniFrac distance for all samples. Each group is represented in a different color and shape. **(A)** Scatterplot from PCoA showed a clear separation of diarrheal (D) from healthy (H) samples (*p* < 0.05, analysis of similarity (ANOSIM)); Principal coordinates 1 and 2 (PC1 and PC2) represent 18.99 and 11.61% of the variance, respectively (x and y axes). **(B)** PCoA scatterplot identified significant bacteria compositional difference among the three ages (*p* < 0.05, ANOSIM). Principal coordinates 1 and 2 (PC1 and PC2) represent 18.4 and 15.55% of the variance, respectively (*x* and *y* axes).

Beta diversity of each group was calculated through principal coordinates analysis (PCoA) based on unweighted UniFrac distances. The PCoA scatterplot revealed clear clustering of gut bacterial communities under PEDV infection (Figure 2A). This pattern was further confirmed by the analysis of similarity (ANOSIM), revealing that gut microbiota differed significantly between the compared groups (*p* < 0.05). Piglets with PEDV showed distinct dynamic changes in their gut bacterial communities, despite being infected with the same virus (*p* < 0.05, ANOSIM) (Figure 2B).

### Characterization of Gut Microbiota in Healthy and PEDV-Infected Piglets

**FIGURE 3:**
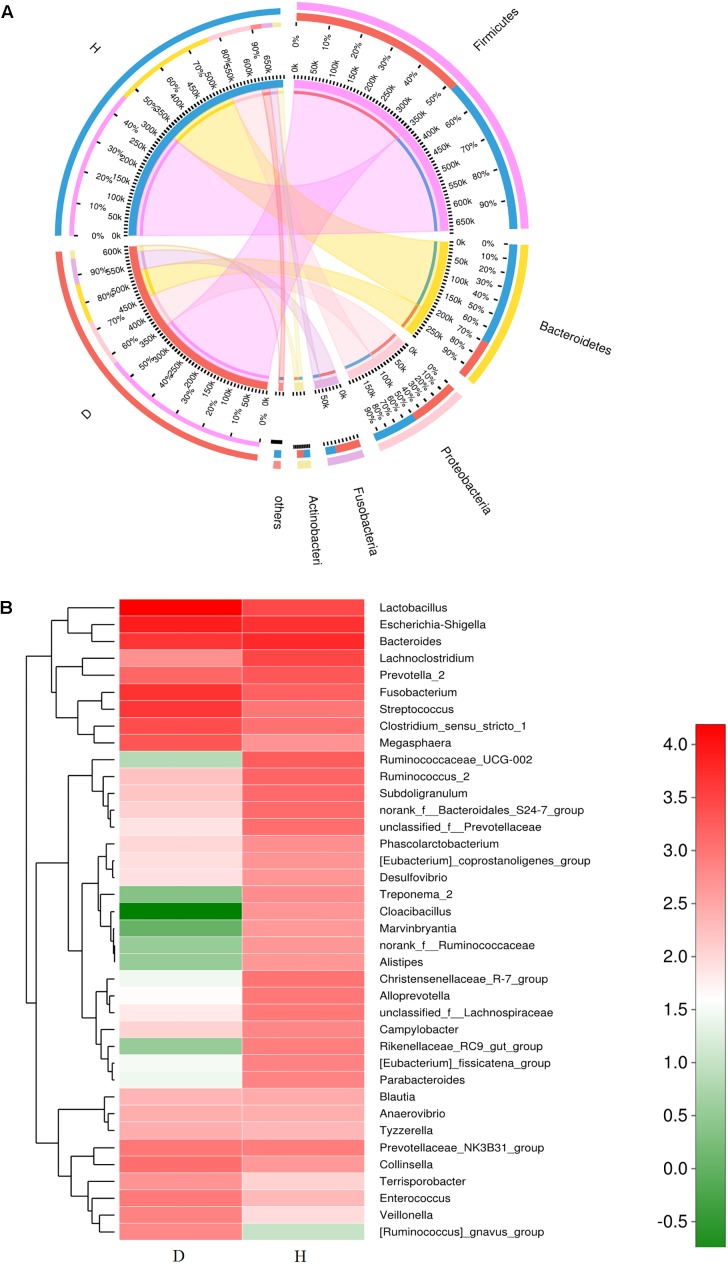
Relative abundance of sequences at the phylum and genus levels in diarrheal (D) and healthy (H) groups. **(A)** Collinear relation diagram between samples and bacterial phylum. **(B)** Heatmap analyses of abundant genera in each group. The heatmap plot depicts the relative percentage of each bacterial genus (variables clustering on the vertical-axis) within each group (horizon-axis clustering). The color of the spots in the right panel represents the relative values (lg) of the genera in each group.

Five dominant phyla were identified in the bacterial communities of all samples. On average, these consisted of over 1% of the total sequences (Figure 3A). The abundance of dominant phyla in healthy piglets was 46.5% for *Firmicutes*, 31.4% for *Bacteroidetes*, 13.0% for *Proteobacteria*, 3.4% for *Fusobacteria*, and 2.5% for *Actinobacteria*, whereas their abundance in diarrheal piglets was 56.4, 13.9, 16.0, 10.8, and 2.9%, respectively (Figure 3A). Only the abundance of *Bacteroidetes* was significantly less in PEDV-infected piglets compared to that in the healthy piglets (*p* < 0.01) (Supplementary Figure S3A).

A total of 267 genera were identified in the gut bacterial communities of healthy piglets compared to 301 genera in diarrheal piglets. In the healthy group, the most abundant bacterial genera were *Bacteroides* (11.24%), *Escherichia-Shigella* (9.51%), *Lachnoclostridium* (5.95%), *Lactobacillus* (5.65%), *Prevotella*_2 (3.99%), *Fusobacterium* (3.26%), and *Ruminococcaceae*_UCG-002 (3.61%) (Figure 3B). In the diarrhea group, *Lactobacillus* (26.79%), *Escherichia-Shigella* (13.99%), *Fusobacterium* (10.45%), *Bacteroides* (7.81%), *Streptococcus* (7.60%), *Lachnoclostridium* (1.45%), and *Clostridium_sensu_stricto*_1 (4.66%) were the predominant genera (Figure 3B). The percentages of *Lactobacillus*, *Escherichia-Shigella*, *Enterococcus*, and *Veillonella* in the diarrhea group were significantly higher compared to the healthy group (*p* < 0.05) (Supplementary Figure S3B).

### Dynamic Change of Gut Microbiota in Diarrheal Piglets During Three Ages Groups

**FIGURE 4:**
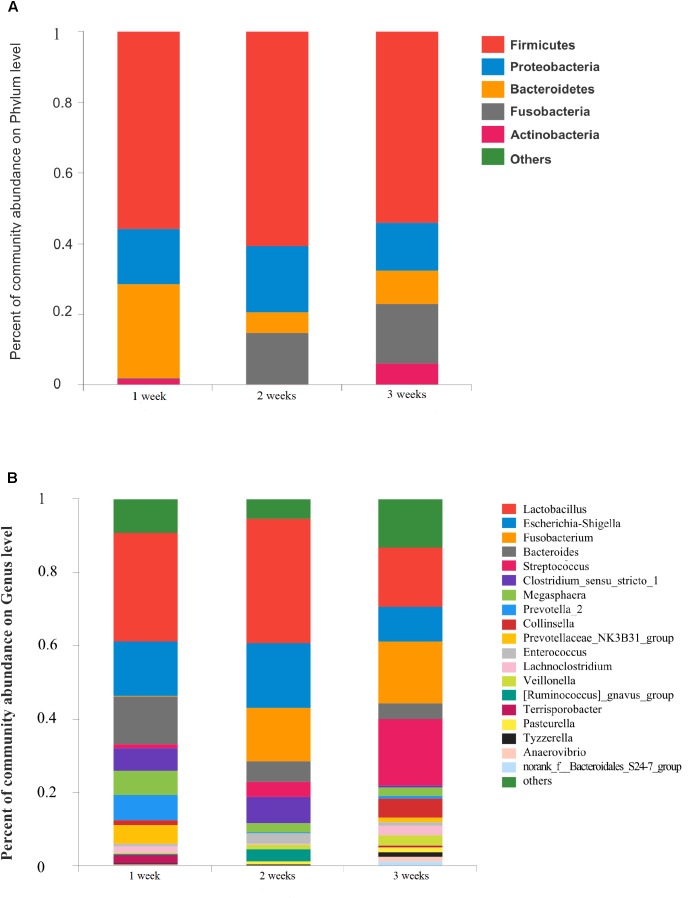
Community bar-plot analysis shows relative abundance of sequences at **(A)** phylum and **(B)** genus levels in the three age groups. OTUs comprising less than 1% of the total abundance at both phylum and genus level are represented.

The gut microbiota in diarrhea samples collected at 3 different ages were characterized to evaluate variability. Proportional abundance was used to identify differentially abundant phyla among groups (Figure 4A). *Firmicutes* was the predominant phylum found in all development stages. Dynamic change was mainly associated with a significant increase in *Veillonella* and *Streptococcus*, and a decrease in *Megasphaera* and *Clostridium_sensu_stricto*_1. A greater abundance of *Bacteroidetes* at 1 week due to the relative abundances of *Bacteroides* and *Prevotella*_2 decreased with age. The significant increase in *Fusobacteria* with age corresponded to a higher abundance of *Fusobacterium* (*p* < 0.05) (Supplementary Figure S4).

The number of genera present in the 1, 2, and 3 weeks groups were 190, 177, and 247, respectively. A total of 112 genera were shared by the 3 different age groups. The dominant genera of *Prevotellaceae_NK3B31_group* and *[Ruminococcus]_gnavus_group* were not identified in the 2 and 3 weeks groups, respectively (Supplementary Figure S4B). In the 1 week group, *Lactobacillus* (29.81%), *Escherichia-Shigella* (14.74%), *Bacteroides* (13.19%), *Prevotella_2* (6.90%), and *Megasphaera* (6.51%) were the predominant genera. We observed a statistically significant predominance of *Lactobacillus* (34.0%), *Escherichia-Shigella* (17.66%), *Fusobacterium* (14.58%), *Clostridium_sensu_stricto*_1 (6.96%), and *Bacteroides* (5.65%) in the 2 weeks group. The prevalent genera found in the 3 weeks group were *Streptococcus* (17.96%), *Fusobacterium* (16.92%), *Lactobacillus* (16.00%), and *Escherichia-Shigella* (9.37%) (Figure 4B). Notably, the relative abundance of *Fusobacterium* and *Veillonella* showed a remarkable increase with age in the diarrheal piglets (*p* < 0.05) (Supplementary Figure S4A).

### Differences in the OTU-Level Phylogenetic Core of Healthy and PEDV-Infected Piglets

The core microbiota was defined as those OTUs with more than 1% relative abundance and present in all healthy samples. The core microbiota of healthy piglets and the relative abundance of those genera in diarrheal piglets are shown in Table 1. *Escherichia-Shigella* and *Streptococcus* were predominant in the diarrhea group. Table 2 indicated the dynamic changes of these 12 core genera in the PEDV-infected piglets. Especially, *Ruminococcaceae_UCG-002* was not identified in the 1 week group. *Rikenellaceae_RC9_gut_group* and *Butyricimonas* were absent in the 2 weeks group, while *Alistipes* was unique to the 3 weeks group.

**Table 1:** Comparisons of 12 core genera between healthy (H) and diarrhea (D) groups.

Phylum	Family	Genus	Mean Relative Abundance (100%)		*p-*values
			H	D	
*Bacteroidete*	*Bacteroidaceae*	*Bacteroides*	11.24	7.81	0.2035
	*Prevotellaceae*	*Prevotella_2*	3.99	2.67	0.03649^∗^
		*Alloprevotella*	1.97	0.19	0.005229^∗∗^
	*Ruminococcaceae*	*Ruminococcaceae_UCG-002*	3.03	0.08	0.000003439^∗∗∗^
	*Rikenellaceae*	*Rikenellaceae_RC9_gut_group*	1.61	0.01	0.000006822^∗∗∗^
		*Alistipes*	1.05	0.01	0.000004826^∗∗∗^
	*Porphyromonadaceae*	*Parabacteroides*	1.49	0.22	0.000021^∗∗∗^
		*Butyricimonas*	1.02	0.02	0.000003373^∗∗∗^
*Firmicutes*	*Streptococcaceae*	*Streptococcus*	1.98	7.60	0.1933
	*Lachnospiraceae*	*Lachnoclostridium*	5.95	1.45	0.01586^∗^
*Proteobacteria*	*Enterobacteriaceae*	*Escherichia-Shigella*	9.51	13.99	0.0135^∗^
	*Desulfovibrionaceae*	*Desulfovibrio*	1.01	0.24	0.0005906^∗∗∗^

*^∗^p < 0.05; ^∗∗^p < 0.01; and ^∗∗∗^p < 0.001.*

**Table 2:** Dynamic changes of 12 core genera content in PEDV-infected piglets.

OUT ID	Mean Relative Abundance (100%)		
	1 week	2 weeks	3 weeks
*Bacteroides*	12.18	6.2	4.478
*Prevotella_2*	67.44	0.23	0.76
*Alloprevotella*	0.1	0.01	0.49
*Ruminococcaceae_UCG-002*	0	0.02	0.24
*Rikenellaceae_RC9_gut_group*	0.01	0	0.04
*Alistipes*	0	0	0.07
*Parabacteroides*	0.03	0.01	0.63
*Butyricimonas*	0.01	0	0.07
*Streptococcus*	1.11	4.32	16.43
*Lachnoclostridium*	1.67	0.16	2.59
*Escherichia-Shigella*	14.66	17.17	9.84
*Desulfovibrio*	0.14	0.03	0.59

### Comparisons Between Healthy and PEDV-Infected Piglets by Functional Investigations

**FIGURE 5:**
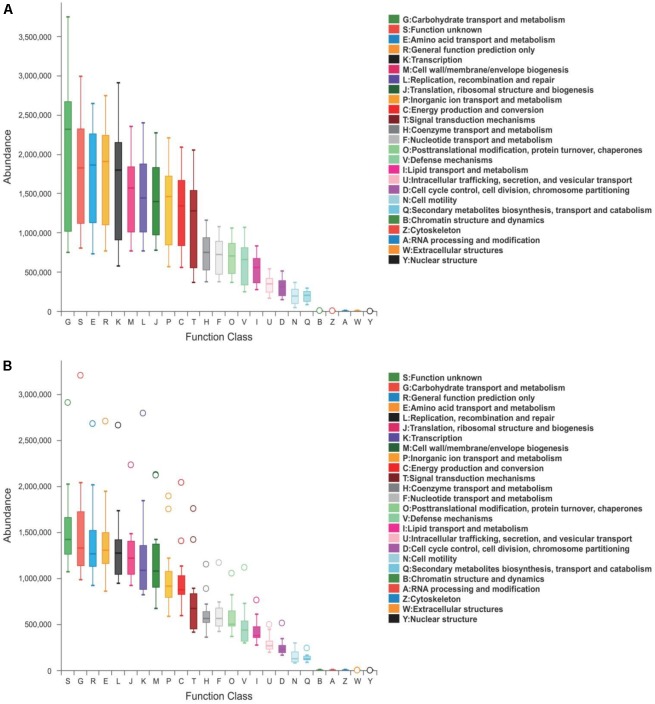
COG functional classification in **(A)** healthy and **(B)** diarrhea piglets. Boxes represent the interquartile range (IQR) between the first and third quartiles. The lines and spots inside the boxes represent the median and mean, respectively. Whiskers denote the lowest and highest values within 1.5 × IQR from the first and third quartiles, respectively. The ring above the box indicates the outliers.

Predicted proteins were functionally categorized based on Cluster of Orthologous Groups (COG) assignment and the abundances in each category were displayed in Figure 5. Among these COG categories, the cluster for “carbohydrate transport and metabolism” represented the largest group in healthy piglets, followed by “function unknown” and “amino acid transport and metabolism” clusters. While most abundance in diarrheal piglets was assigned to the “function unknown” category, followed by “carbohydrate transport and metabolism” and “general function prediction only.” The statistical difference between COG predicted functional abundance of healthy and diarrhea groups is described in Table 3. When challenged with PEDV infection, the functional categories of suckling piglets showed marked changes, including “carbohydrate transport and metabolism,” “amino acid transport and metabolism,” “energy production and conversion,” “defense mechanisms,” and “lipid transport and metabolism” (*p* < 0.05).

**Table 3:** Comparisons of COG functional abundance between healthy (H) and diarrhea (D) groups.

Function code	Description	Median abundance		*p*-value
		H	D	
G	Carbohydrate transport and metabolism	2037230	1512602	0.00951^∗∗^
S	Function unknown	1789903	1510502	0.0678
E	Amino acid transport and metabolism	1727646	1377096	0.0365^∗^
R	General function prediction only	1725785	1386180	0.0507
K	Transcription	1646558	1212702	0.0502
M	Cell wall/membrane/envelope biogenesis	1486729	1166449	0.0734
L	Replication, recombination and repair	1449998	1308059	0.1677
J	Translation, ribosomal structure and biogenesis	1411434	1251860	0.0723
P	Inorganic ion transport and metabolism	1315868	990624.3	0.0513
C	Energy production and conversion	1283908	959898.9	0.0492^∗^
T	Signal transduction mechanisms	1125981	722264.6	0.0501
H	Coenzyme transport and metabolism	745593.3	600065.6	0.00529^∗∗^
F	Nucleotide transport and metabolism	691720.9	595203.4	0.00883^∗∗^
O	Posttranslational modification, protein turnover, chaperones	688619.6	566269.2	0.00682^∗∗^
V	Defense mechanisms	602100.1	471338.2	0.00349^∗∗^
I	Lipid transport and metabolism	523449.2	416518.3	0.00721^∗∗^
U	Intracellular trafficking, secretion, and vesicular transport	336953	283189.2	0.0629
D	Cell cycle control, cell division, chromosome partitioning	301444.1	24585.71	0.0506
N	Cell motility	194057.2	142038.9	0.0612
Q	Secondary metabolites biosynthesis, transport and catabolism	187739.7	137872.2	0.0785
B	Chromatin structure and dynamics	2972	1926.056	0.0663
Z	Cytoskeleton	1934.6	1056	0.0623
A	RNA processing and modification	998.35	1241.056	0.0734
W	Extracellular structures	149.3	348.9	0.00236^∗∗^
Y	Nuclear structure	2.5	0.11	0.00591^∗∗^

*^∗^p < 0.05 and ^∗∗^p < 0.01.*

## Discussion

This study showed a significantly decreased alpha diversity in the gut bacterial population of PEDV-infected piglets compared to the controls. Meanwhile, alpha diversity in the gut microbiota of diarrheal piglets showed a continuous increase with age. High diversity is considered to be indicative of mature gut microbiota, which is less sensitive to environmental factors and less vulnerable to disturbances (Chen et al., 2017). Dynamic changes in the gut microbiota of piglets may explain decreased morbidity and mortality observed in older PEDV-infected piglets (Alonso et al., 2014).

In our study, *Escherichia-Shigella*, *Enterococcus*, *Fusobacterium*, and *Veillonella* were markedly increased in the diarrhea group. Among these, no statistical significance was observed between changes in age related abundance of *Escherichia-Shigella* and *Enterococcus* in PEDV-infected piglets. There is evidence that *Escherichia coli* and *Enterococcus* spp., which are commonly present in piglet gastrointestinal tract, are both potentially pathogenic bacteria awaiting potential opportunities to become pathogenic (Jonach et al., 2014). *E. coli* has been reported to be associated with enteritis and diarrhea in suckling piglets (Song et al., 2017). Certain members of *Enterococcus* may cause diarrhea in neonatal piglets (Jonach et al., 2014). However, whether the proliferation of these potential pathogens following PEDV infection, has a persistent negative impact on piglets warrants further research.

Another two genera, *Fusobacterium* and *Veillonella*, exhibited a statistically significant increase in age related abundance in diarrhea piglets (*p* > 0.05). Growing evidence suggests that *Fusobacterium* is closely correlated with cancer in humans and other diseases in animals (De Witte et al., 2017; Yang et al., 2017). *F. nucleatum*, in particular, may play a vital role in the pathogenesis of various diseases of the gut, including intestinal inflammation and colon cancer (Rubinstein et al., 2013; Jia et al., 2017). Reportedly, as gut microbiota matured, the abundance of *Fusobacterium* decreased sharply from suckling period to weaning period in healthy piglets (Chen et al., 2017). In addition, *Veillonella*, as early colonizers, can coaggregate with many bacteria, including *F. nucleatum*, during various stages of oral biofilm formation (Zhou et al., 2017). It has been reported that *Veillonella* species may contribute to oral dysbiosis in inflammatory bowel disease (IBD) patients (Said et al., 2014). Parallel to that seen in our study, *Veillonella* also exhibited a significant increase with age in young children with moderate-to-severe diarrhea in some countries (Pop et al., 2014). The specific mechanism underlying these distinct age-related shifts in *Fusobacterium* and *Veillonella* during PEDV infection remains unclear.

Interestingly, we observed statistically significant predominance of *Lactobacillu*s, especially that of *Lactobacillus johnsonii*, in the diarrhea groups (*p* < 0.0001). *Lactobacillus* are commonly investigated as probiotic agents, which influence host immunity and disease susceptibility (Niederwerder, 2017). *L. johnsonii* is known to fortify the cell against an *E. coli* challenge through tight junction protein modulation and direct interaction with the pathogens (Liu H.Y. et al., 2015). Recent research has shown that *L. johnsonii* BS15 can be applied as a probiotic to control diarrhea in piglets (Xin et al., 2019). Similar to our study, *Lactobacillus* was increased in children diagnosed with irritable bowel syndrome when compared with a healthy group (Rigsbee et al., 2017). Whether the increase of *Lactobacillus* is protective against PED, through its effect on pathogen inhibition or host immunomodulation, requires further evaluation.

Notably, the relative abundance of SCFA-producing bacteria included in the core microbiota decreased following PEDV infection. *Bacteroides* and *Alloprevotella* primarily produce succinate and acetate (Shah and Collins, 1989; Downes et al., 2013). *Ruminococcus* ferments carbohydrates and produces acetate and propionate (Park et al., 2012). In addition, *Prevotella*, *Rikenellaceae, Alistipes*, and *Butyricimonas* are predominantly SCFA-producing genera (Chen et al., 2017; Qing et al., 2018). SCFAs, which are major anions found in the gut, are rapidly absorbed by colonic epithelial cells (Chen et al., 2017). Compelling evidence indicates that these small molecules protect the host against colonic diseases, exhibit anti-inflammatory effects and promote energy intake by intestinal fibers (Maslowski et al., 2009; De Filippo et al., 2010; Fukuda et al., 2011). An observable decrease in SCFA levels in PEDV-infected piglets indicated that these molecules may play a key role in compromising intestinal and immune system homeostasis.

The core genera are shared by all healthy piglets, however, *Ruminococcaceae_UCG-002* and *Alistipes* were not identified in the gut bacterial communities of diarrheal piglets at 1 week of age. *Alistipes* are known producers of anti-inflammatory metabolites, which promote the differentiation of anti-inflammatory Treg/Tr1 cells in the gut (Li et al., 2016). Moreover, higher abundance of the *Ruminococcaceae* family may enable higher energy harvesting in suckling piglets which is an adequate prevention strategy for pathogen infection (Dou et al., 2017). Similarly, 3 core genera (*Rikenellaceae_RC9_gut_group*, *Alistipes*, and *Butyricimonas*) were not identified in the 2 weeks group. Correlation analysis of intestinal flora and biochemical factors indicated a significantly positive correlation between *Rikenellaceae* and anti-inflammatory cytokines (Tao et al., 2017). In addition, a reduction in *Butyricimonas* has been noted in numerous autoimmune and inflammatory diseases including IBD, rheumatoid arthritis and type 1 diabetes (Scher et al., 2013; de Goffau et al., 2014; Wang et al., 2014). Considered together, the differences in gut microbiota that were observed when challenged with PEDV infection may be associated with the age of piglets. Therefore, providing different supplements to suckling piglets at different ages according to the deficiency of beneficial bacteria at each age, may prevent or alleviate PEDV-infection-induced diarrhea.

Many functions of microorganisms are performed in cooperation with bacteria in the intestinal microbial ecosystem (Huang et al., 2018). Certain SCFA-producers are considered to be outstanding contributors to carbohydrate fermentation, mucosal defense mechanisms, adipogenesis and lipid oxidation (Hooper et al., 2002; Liu et al., 2016; Fava et al., 2018). Therefore, decreased abundance of SCFA-producing bacteria following PEDV infection, may partially account for the relatively lower abundance of “carbohydrate transport and metabolism,” “defense mechanisms,” and “lipid transport and metabolism.” A similar finding was reported by a study which indicated that changes in the relative abundances of *Veillonella*, *Lactobacillus*, and *Prevotella* may lead to variation in “energy metabolism,” “amino acid metabolism,” and “biosynthesis of secondary metabolites” in the small intestine of PEDV-infected piglets (Huang et al., 2018). Overall, when challenged with PEDV, distinct changes in gut microbiota ranging from phylum to genus level may disrupt normal physiological functions of the intestine.

## Conclusion

In conclusion, our study unveiled dysbiosis of gut microbiota in PEDV-infected piglets and described age related changes in the gut microbiota of diarrheal piglets. *Escherichia-Shigella*, *Enterococcus*, *Fusobacterium*, and *Veillonella* were significantly increased, while SCFA-producing bacteria, such as *Rikenellaceae_RC9_gut_group*, *Butyricimonas*, and *Alistipes*, were reduced in PEDV-infected piglets. Notably, *Fusobacterium* and *Veillonella* increases remarkably with age in diarrheal piglets. In addition, age related deficiency of beneficial bacteria in diarrheal piglets indicated that age based administration of supplements to maintain stability in the gut microbiota may be a useful strategy to prevent or alleviate PEDV infection.

## Author Contributions

AH, HY, CL, and RC conceived and designed the experiments. AH, HY, CL, RC, and QW performed the experiments. AH, HY, CL, RC, and LS analyzed the data and contributed analysis tools. AH, HY, CL, and RC wrote the manuscript. All authors read and approved the final manuscript.

## Conflict of Interest Statement

The authors declare that the research was conducted in the absence of any commercial or financial relationships that could be construed as a potential conflict of interest.

## Footnotes

1 http://drive5.com/usearch/manual/uchime_algo.html

2 http://drive5.com/uparse/

3 http://rdp.cme.msu.edu/

4 http://circos.ca/

5 http://string-db.org/
