# Supplementary material for: Dynamic Change of Gut Microbiota During Porcine Epidemic Diarrhea Virus Infection in Suckling Piglets
Source: Front Microbiol. 2019 Feb 25;10:322. doi: 10.3389/fmicb.2019.00322 (PMC6397872; doi:10.3389/fmicb.2019.00322)
Supplement: Supplementary file 1 [file Presentation_1.pdf]

Supplementary Material

Dynamic change of gut microbiota during porcine epidemic diarrhea virus infection in suckling piglets

Anni Huang[1*], Rujian Cai[2, 3, 4*], Qun Wang[1], Lei Shi[5], Chunling Li[2, 3, 4**], He Yan[1**]

[1.] *School of Food Science and Engineering, South China University of Technology, Guangzhou 510641, China*

[2.] *Institute of Animal Health Guangdong Academy of Agricultural Sciences, Guangzhou 510640, Guangdong, China*

[3.] *Guangdong Provincial Key Laboratory of Livestock Disease Prevention, Guangzhou, China*

[4.] *Guangdong Open Laboratory of Veterinary Public Health, Guangzhou, China*

[5.] *Institute of Food Safety and Nutrition, Jinan University, Guangzhou 510632, China*

Running title: Gut microbiota of PEDV-infected piglets

*These authors contributed equally

****Corresponding author:**

Dr. Chunling Li, Ph.D.

Mailing address: Institute of Animal Health Guangdong Academy of Agricultural Sciences, Guangzhou 510640, Guangdong, China

E-mail Address: lclclare@163.com (Chunling Li)

Tel: +86-20-85291377

Dr. He Yan, Ph.D.

Mailing address: School of Food Science and Engineering, South China University of Technology, Guangzhou 510640, P.R. China

E-mail Address: yanhe@scut.edu.cn (He Yan)

Tel: +86-20-87113848; fax: +86-20-87112734

Supplemental Figure

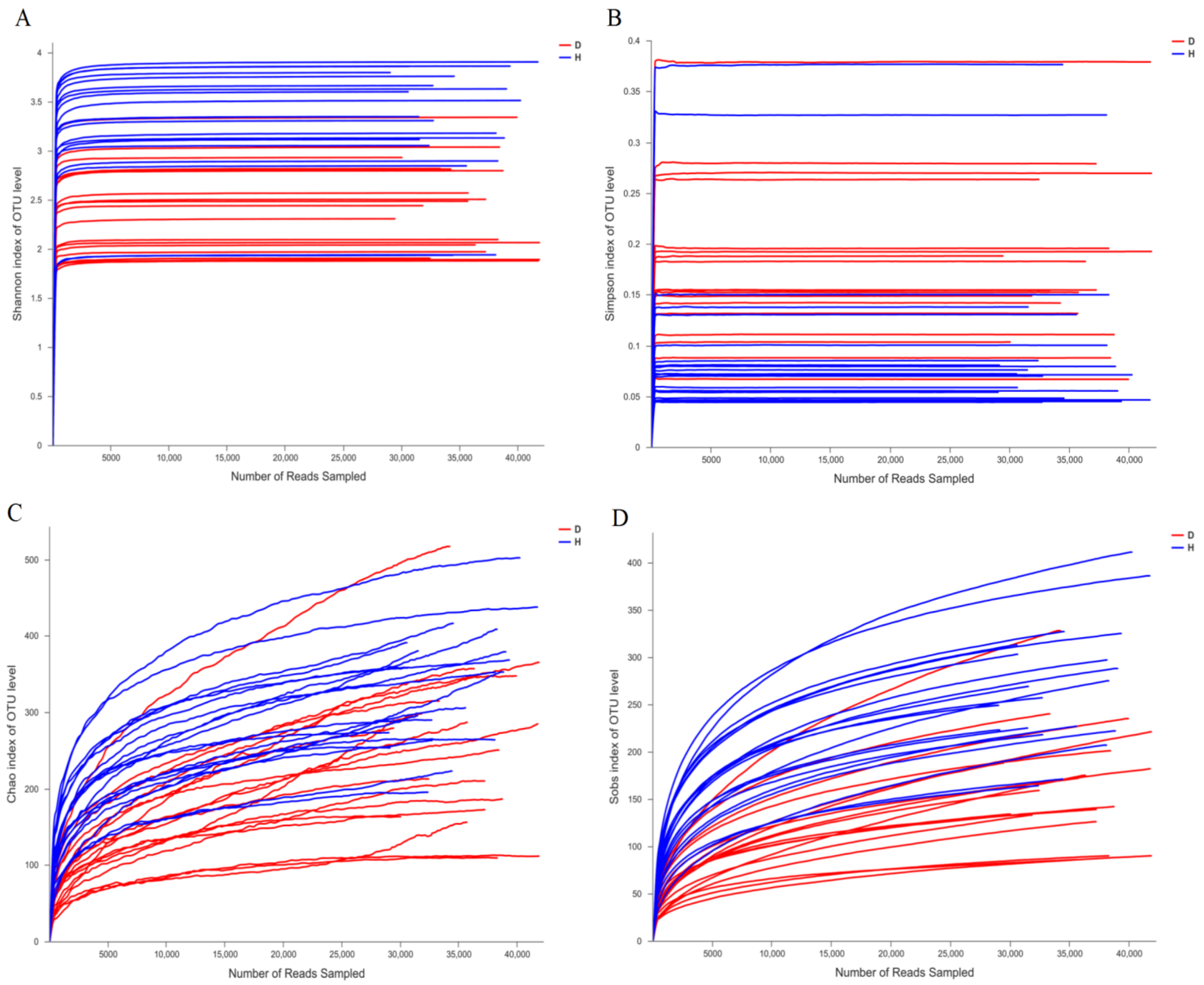

Supplemental Figure 1. Bacterial rarefaction curves based on Shannon index (A), Simpson index (B), Chao index (C) and Sobs index (D) were used to assess the depth of coverage for each healthy (H) and diarrheal (D) sample.

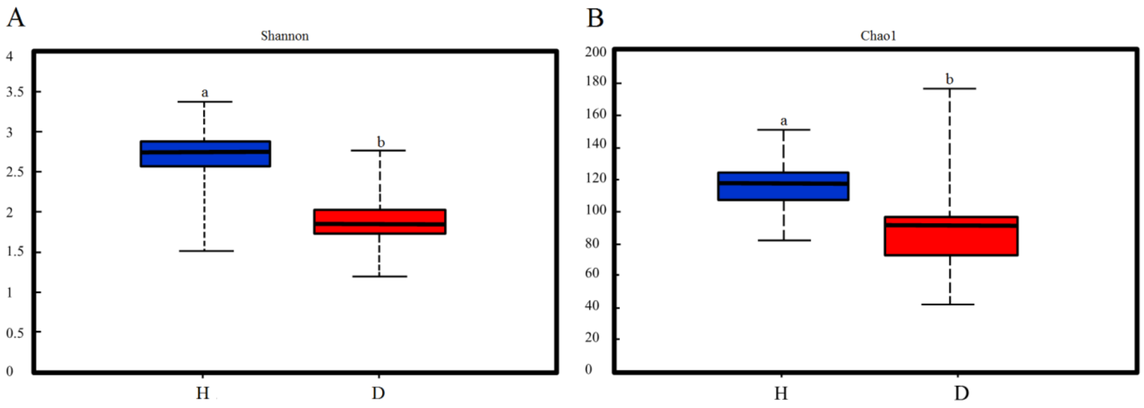

Supplemental Figure 2. Bacterial alpha diversity differences between healthy (H) and diarrheal (D) piglets determined by (A) Shannon index and (B) Chao1 index. Different letters above the bars denote a significantly different alpha diversity index among the groups tested by paired sample Wilcoxon signed-rank test and adjusted by false discovery rate (FDR, $p < 0.05$).

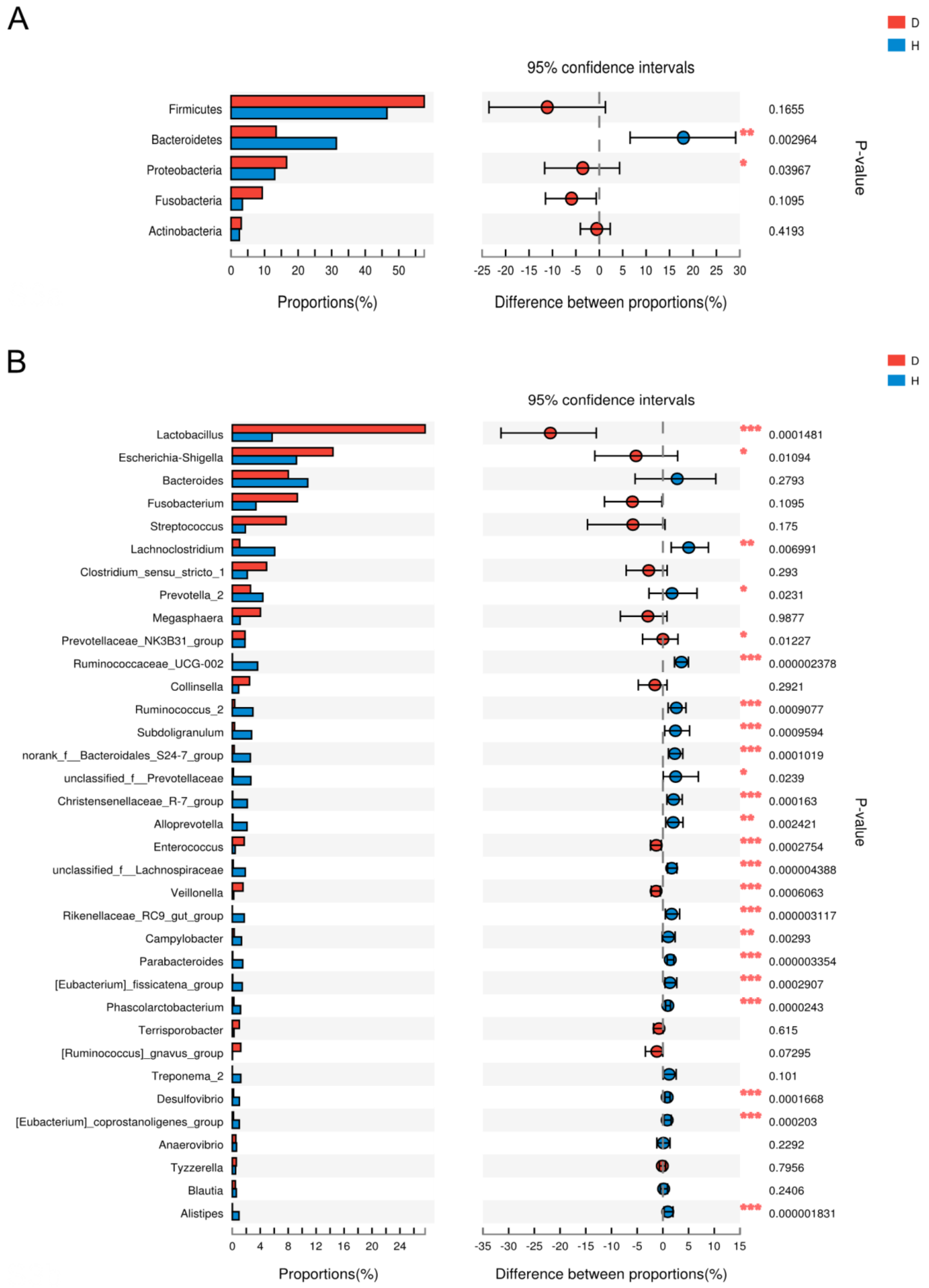

Supplemental Figure 3. Bar graphs illustrate the differences in (A) predominant bacterial phyla and (B) genera between diarrheal (D) and healthy (H) groups. Data of PRD and control groups are shown as relative abundance (%) of genera in each group. Statistical analysis was performed by the Wilcoxon rank-sum test. $* p < 0.05$, $** p < 0.01$, and $*** p < 0.001$.

A

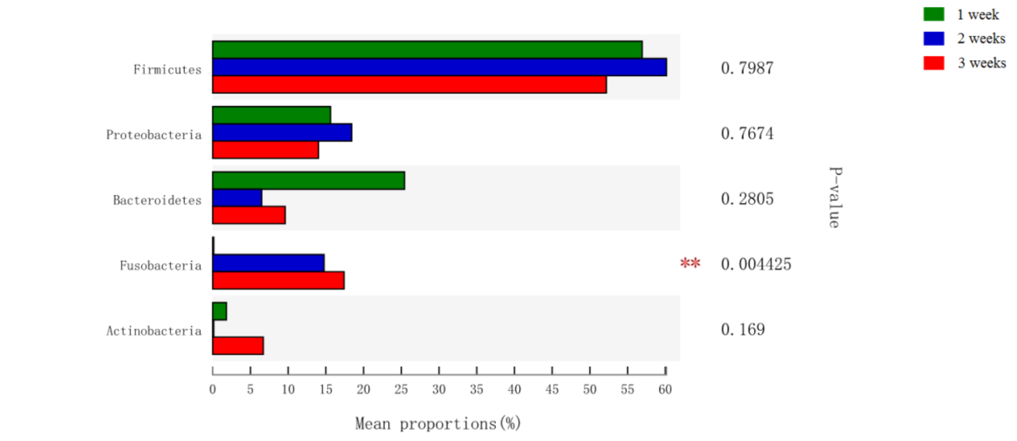

B

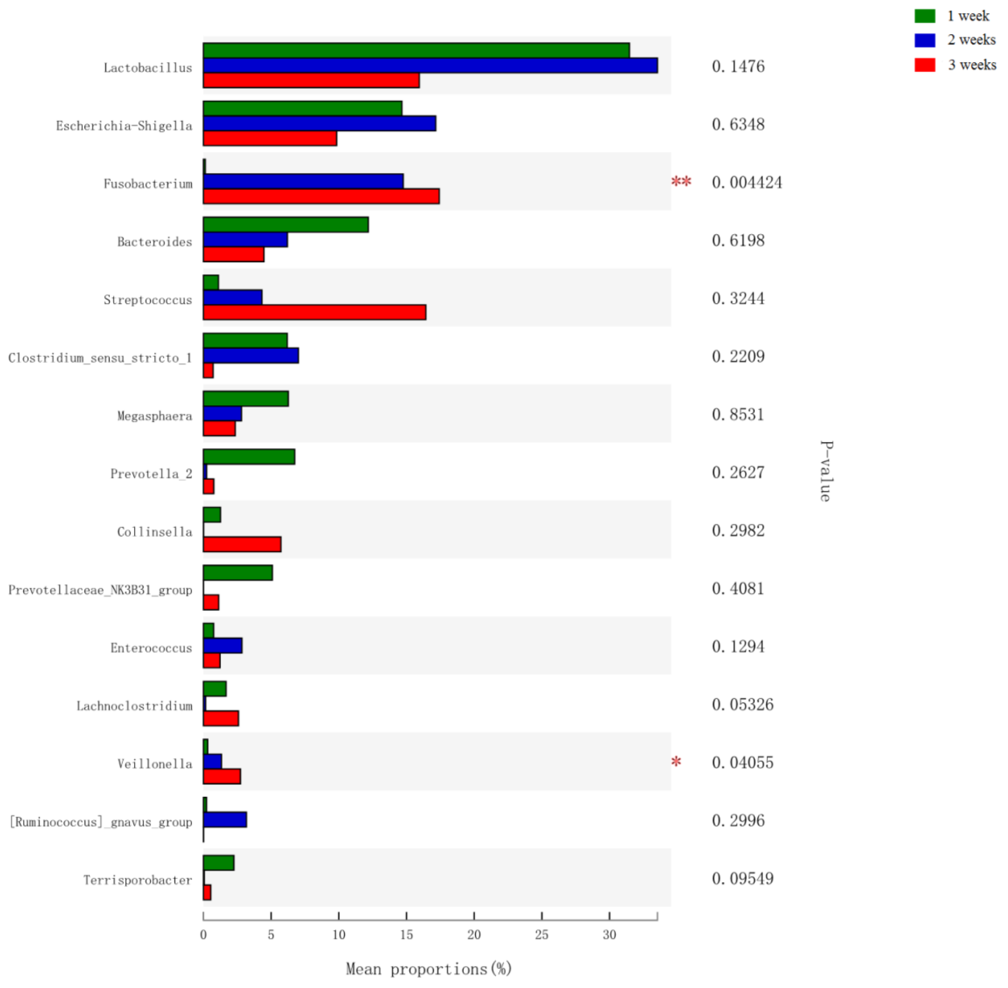

Supplemental Figure 4. Bar graphs illustrate the differences in (A) predominant bacterial phyla and (B) genera among three age groups. Data of PRD and control groups are shown as relative abundance (%) of genera in each group. Statistical analysis was performed by the Wilcoxon rank-sum test. $* p < 0.05$, $** p < 0.01$, and $*** p < 0.001$.
